# Supplementary material for: PD-L1 positive astrocytes attenuate inflammatory functions of PD-1 positive microglia in models of autoimmune neuroinflammation
Source: Nat Commun. 2023 Sep 9;14:5555. doi: 10.1038/s41467-023-40982-8 (PMC10492803; doi:10.1038/s41467-023-40982-8)
Supplement: Supplementary file 1 — Supplementary Information [file 41467_2023_40982_MOESM1_ESM.pdf]

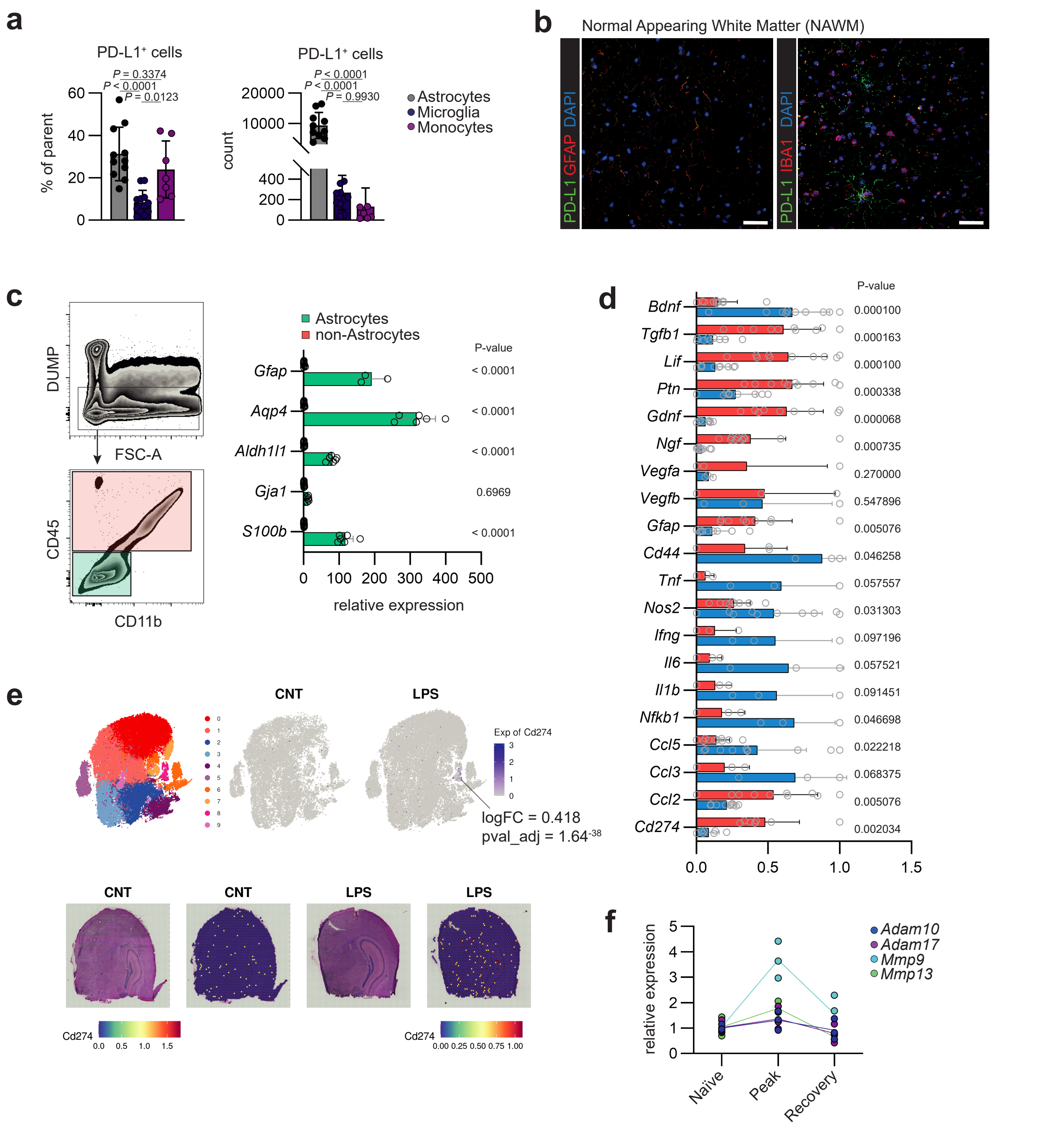

Supplementary Figure 1. Astrocytic expression of PD-L1 in the context of CNS inflammation. a, relative PD-L1 expression (%of parent) and absolute counts by astrocytes (n = 11), monocytes (n = 7), and microglia (n =12) in the inflamed CNS quantified by flow cytometry. **b,** immunohistochemical staining of PD-L1+ astrocytes (GFAP+; left) and microglia (Iba1+; right) in normal appearing white matter (NAWM) from MS patients. 30 µm scale bar. **c,** sorting strategy for astrocytes from CNS and expression of astrocyte markers Gfap, Aqp4, Aldh1l1, Gja1, and S100b by sorted astrocytes (n = 4) and non-astrocytes (n = 10) quantified by RT-qPCR. **d,** normalized relative expression of genes in PD-L1+ and PD-L1- EAE astrocytes. n = 3/10 PD-L1+, n = 3/10 PD-L1-. **e,** tSNE plot (upper) and Visium spatial expression (lower) of Cd274 expression in astrocyte subclusters following peripheral LPS- or vehicle (CONT)-injection obtained from Hasel et al. 86. **f,** mRNA expression of metalloproteases Adam10, Adam17, Mmp9, and Mmp13 in sorted astrocytes at the naïve, peak and recovery stage of EAE. One-way ANOVA with Tukey's multiple comparisons test in **(a)**, unpaired t-test with two-stage step-up FDR control in **(c, d)**. Exact P-values are provided in the figure. Data are shown as mean ± SD.

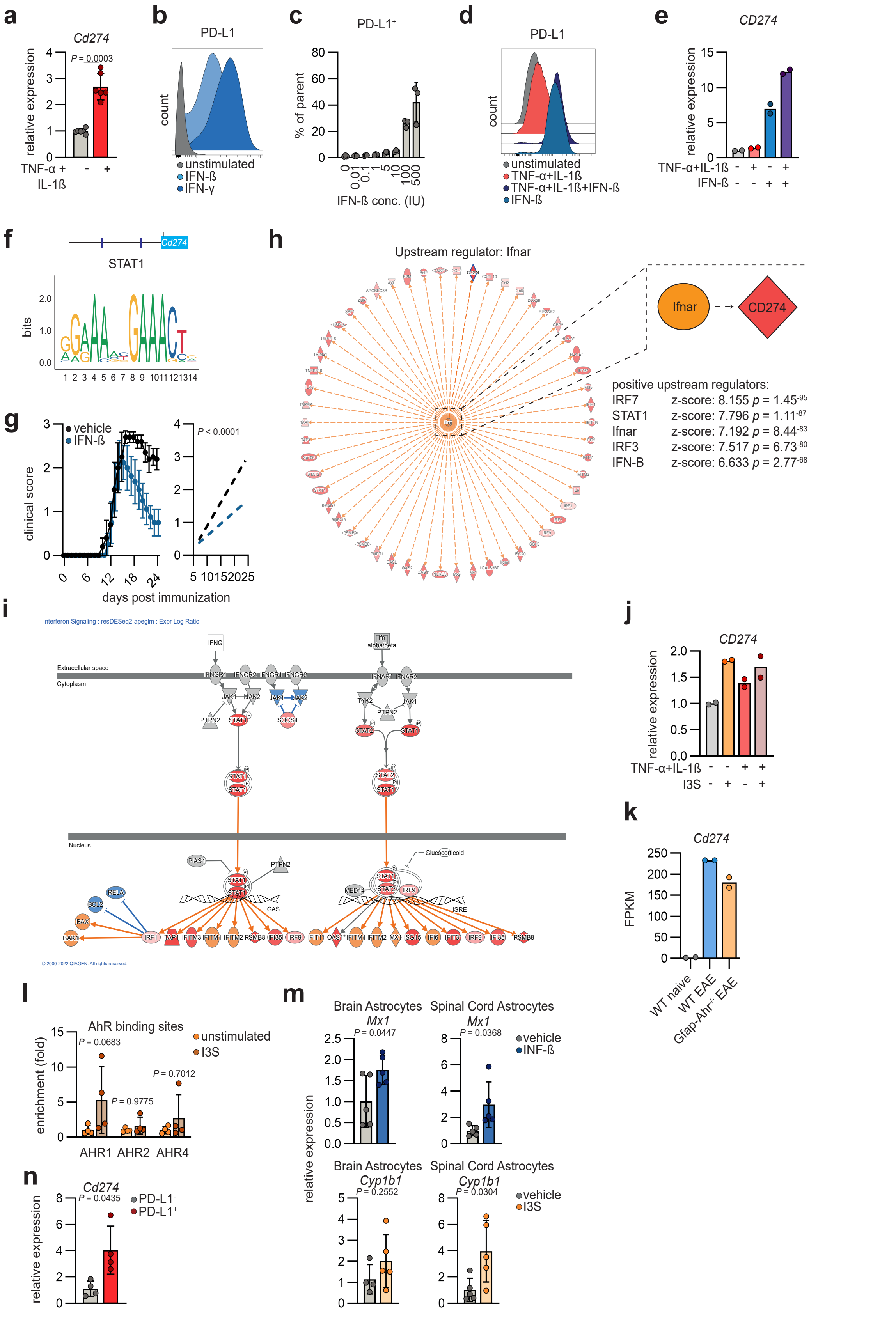

Supplementary Figure 2. Transcriptional control of Cd274 in astrocytes. a, Cd274 expression by primary mouse astrocytes following stimulation with TNF-α and IL-1β or vehicle. n = 5 per group. b, representative scatterplots (entire group concatenated) of PD-L1 expression by primary mouse astrocytes following stimulation with IFN-β, IFN-γ, or vehicle. n = 3 per group. **c,** flow cytometric quantification (% of parent) of PD-L1 expression by primary mouse astrocytes following stimulation with different concentrations of IFN-β. n = 3 per group. **d,** representative scatterplots (entire group concatenated) of PD-L1 expression by human astrocytes following stimulation with TNF-α, IL-1β, and IFN-β. n = 3 per group. **e,** CD274 expression by human astrocytes in response to TNF-α and IL-1β ± IFN-β. n = 2 per group. **f,** predicted STAT1-binding sites in the Cd274 promoter by JASPAR 34. **g,** EAE development in mice intranasally treated with vehicle or IFN-β starting at day 7 post immunization. n = 5 per group. Experiment repeated twice. **h,** Ingenuity Pathway Analysis of the upstream regulatory network in astrocytes derived from EAE mice that received intranasal IFN-β administration. Upregulation in comparison to vehicle treated mice is indicated by red color. **i,** interferon signaling network in astrocytes derived from EAE mice that received intranasal IFN-β administration. Upregulation in comparison to vehicle treated mice is indicated by red color, downregulation by blue color. **j,** CD274 expression by human astrocytes in response to TNF-α and IL-1β ± I3S. n = 2 per group. **k,** Fragments Per Kilobase Million (FPKM) values of sorted astrocytes from WT, WT EAE, and GFAP^{Cre} Ahr$^{fl/fl}$ EAE mice. n = 2 per group. **l,** ChIP-qCPR analysis of AhR recruitment to the Cd274 promoter following stimulation with I3S or vehicle, n = 4 per group. m, Mx1 (IFN-signaling control) and Cyp1b1 (AhR-signaling control) expression by astrocytes obtained from EAE mice intranasally treated with IFN-β or I3S. n = 5 per group. **n,** Cd274 expression by PD-L1+ and PD-L1- sorted astrocytes. n = 4 per group. Unpaired t-test (two-sided) in **(a, m, n)**, Linear regression starting day 7 p.i. in **(g)**, One-way ANOVA with Dunnett's multiple comparisons test in **(f)**, Two-way ANOVA with Sidak's multiple comparisons test in **(l)**. Exact P-values are provided in the figure. Data are shown as mean ± SEM in **(g)**. Data are shown as mean ± SD if not indicated otherwise.

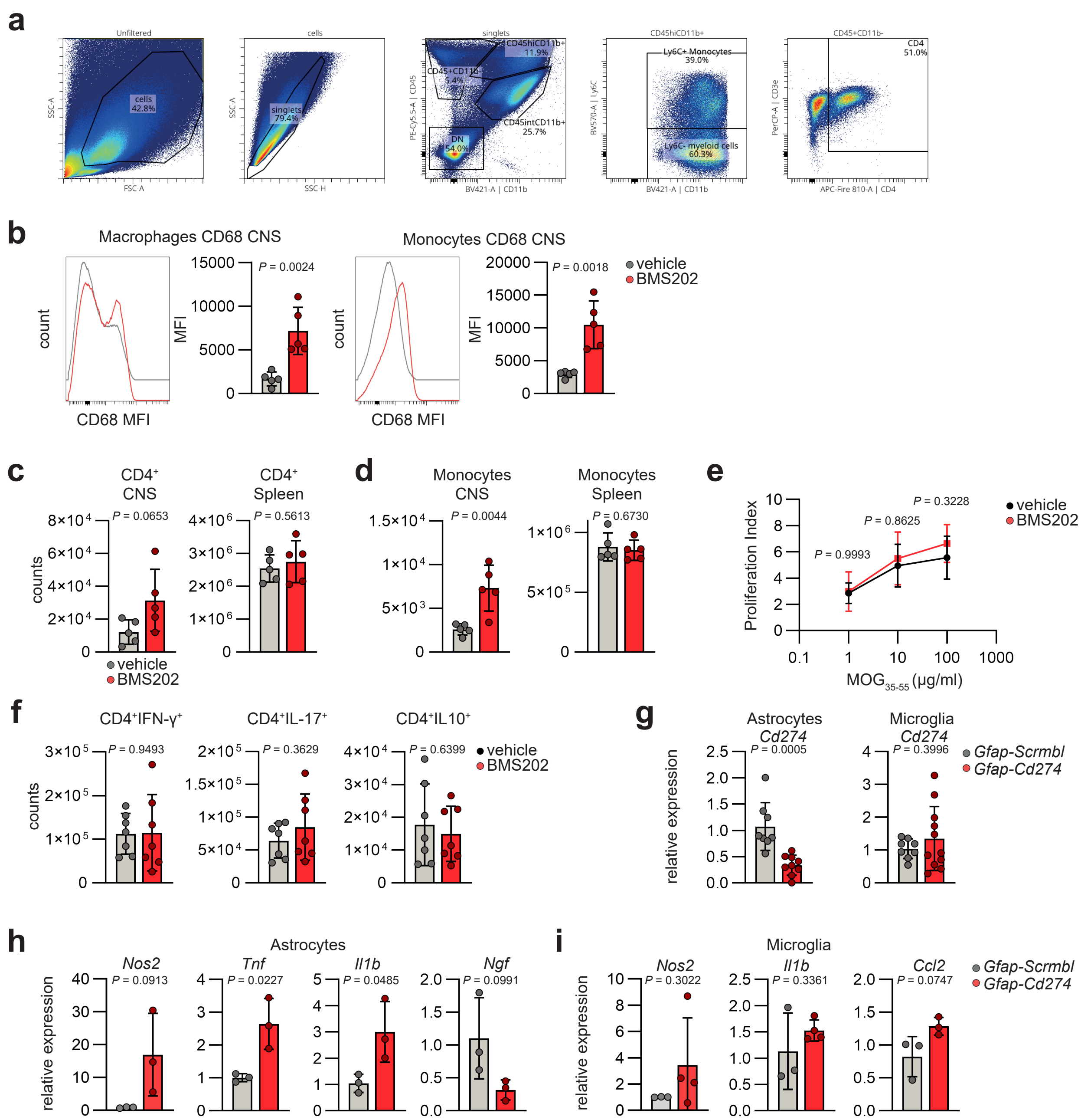

Supplementary Figure 3. Astrocyte-derived PD-L1 attenuates neuroinflammation. a, gating strategy used identify Ly6C+ monocytes and CD4+ T cells in the CNS (and spleen). **b,** representative scatter plots and median-fluorescence intensity (MFI) quantification of CD68 expression by macrophages and monocytes in the CNS of vehicle or BMS202-treated mice. n = 5 per group. **c,** counts of CD4+ T cells in the CNS and spleen of BMS202 or vehicle-treated mice. n = 5 per group. **d,** counts of Ly6C+ monocytes in the CNS and spleen of BMS202 or vehicle-treated mice. n = 5 per group. **e,** proliferation index of splenic CD4+ T cells obtained from BMS202 or vehicle-treated mice that were challenged with varying concentrations of MOG35-55 in vitro. n = 9 per group. **f,** counts of TH1 ($CD45^+CD11b^-CD4^+IFN\text{-}\gamma^+$), TH17 ($CD45^+CD11b^-CD4^+IL\text{-}17^+$), and regulatory $IL\text{-}10^+CD4^+$ T cells in spleens of vehicle or BMS202 treated mice. n = 7 per group. **g,** Cd274 expression in FACS-sorted astrocytes and microglia from Gfap-Scrmbl (n = 8) and Gfap-Cd274 EAE mice (n = 9/11). **h,** mRNA expression of *Nos2*, *Tnf*, *Il1b*, *Ngf* by astrocytes (Gfap-Scrmbl n = 3; Gfap-Cd274 n = 3) **(h)** and *Nos2*, *Il1b*, *Ccl2* by microglia (Gfap-Scrmbl n = 3; Gfap-Cd274 n = 3/4) **(i)** from Gfap-Scrmbl and Gfap-Cd274 EAE mice. Two-way ANOVA with Sidak's multiple comparisons test in **(e)**, unpaired t-test (two-sided) if not indicated otherwise. Exact P-values are provided in the figure. Data are shown as mean ± SD in if not indicated otherwise.

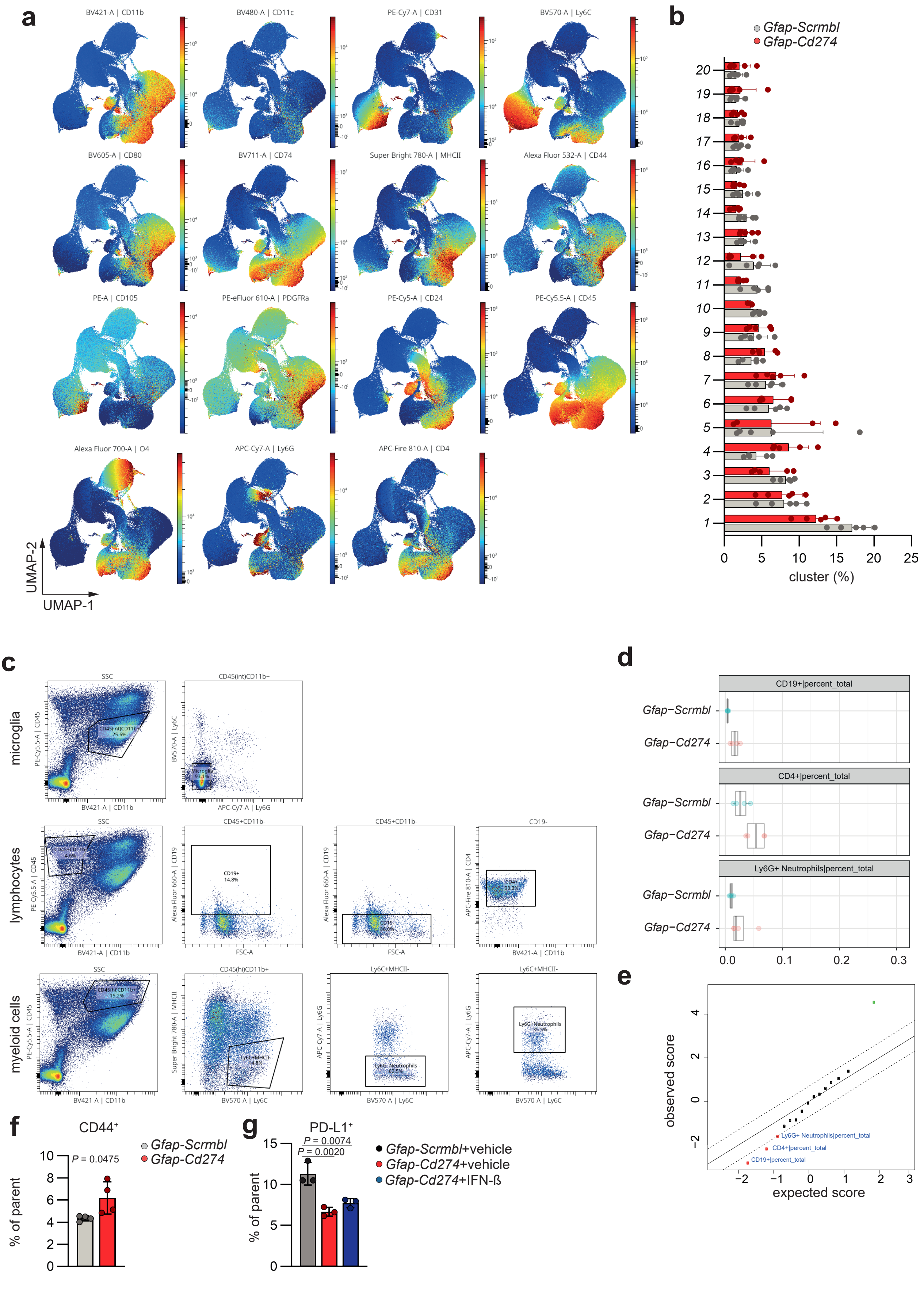

Supplementary Figure 4. Astrocyte-derived PD-L1 regulates CNS inflammation. a, UMAP plots of CNS cells analyzed by high-dimensional flow cytometry in Gfap-Scrmbl (n = 5) and Gfap-Cd274 (n = 5) mice. The color represents the intensity of the respective surface marker (red indicates high expression, blue indicates low expression). **b,** relative abundance (% of singlets) of FlowSOM clusters in the CNS of Gfap-Scrmbl (n = 5) and Gfap-Cd274 (n = 5) mice analyzed by high-dimensional flow cytometry. **c,** representative gating strategy for microglia, lymphocytes, myeloid cells and their respective subsets. **d,** SAM (Statistical Analysis of Microarray) 78 boxplots and SAM-plot **(e)** of cell population abundances in the CNS that were significantly different between Gfap-Scrmbl and Gfap-Cd274 mice. n = 4 per group. **f,** CD44 expression (% of parent) by microglia in Gfap-Scrmbl and Gfap-Cd274 mice during EAE. n = 4 per group. g, flow cytometric quantification of PD-L1 expression by astrocytes in Gfap-Scrmbl and Gfap-Cd274 mice that were intranasally treated with vehicle or IFN-β. n = 3 per group. One-way ANOVA with Tukey's multiple comparisons test in **(g)**, unpaired t-test in if not indicated otherwise. Data are shown as mean ± SD in if not indicated otherwise. Data are shown as mean with the 25th and 75th percentiles in **(d)**.

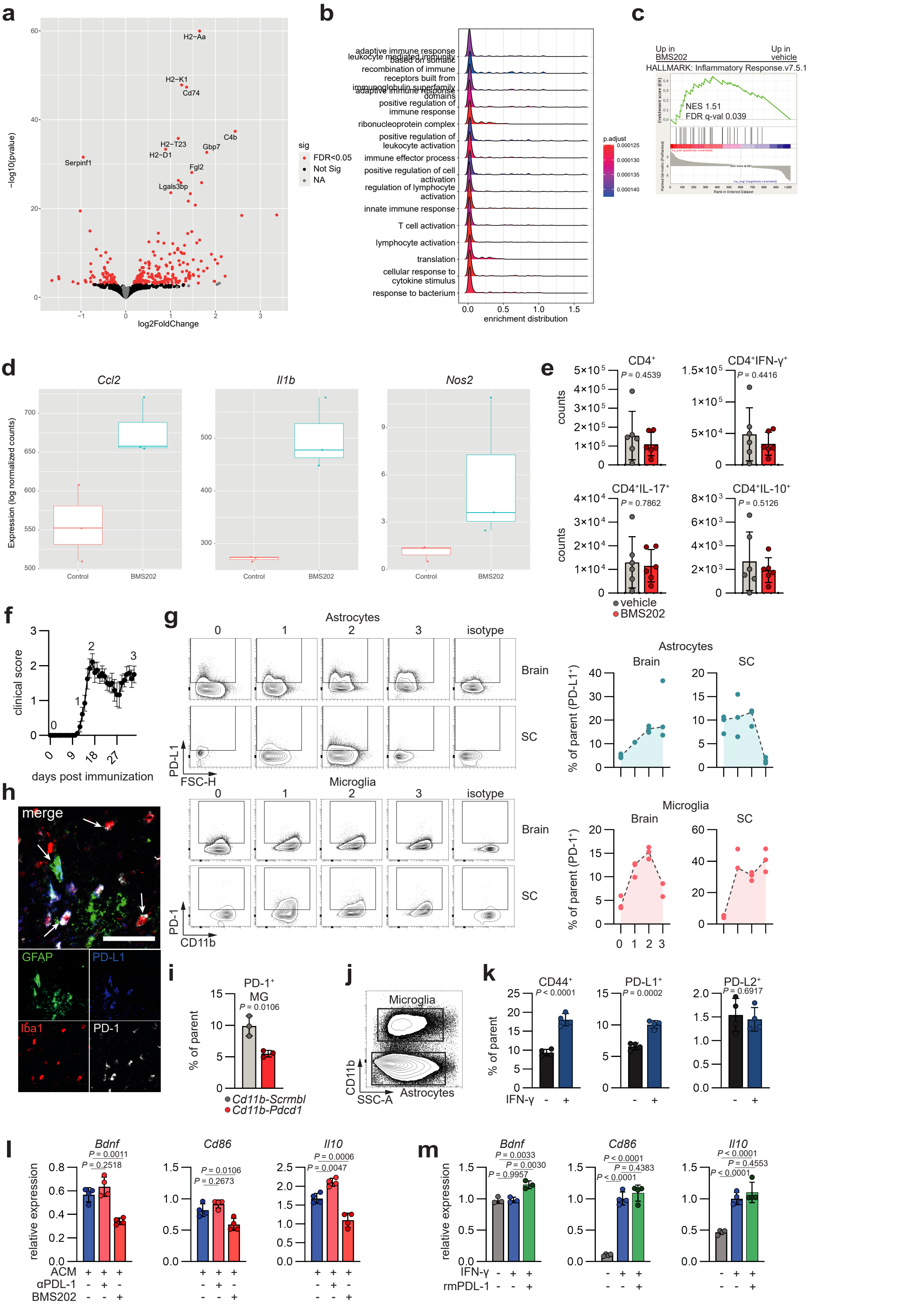

Supplementary Figure 5. PD-L1 / PD-1 signaling modulates glial responses during chronic neuroinflammation. a, volcano plot of genes differentially regulated in microglia derived from NOD/ShiLtJ treated with BMS202 or vehicle during the progressive phase of EAE. b, pathway enrichment and GSEA **(c)** of astrocytic gene expression from NOD/ShiLtJ mice treated with vehicle or BMS202. n = 3 per group. **d,** log normalized counts of *Ccl2*, *Il1b*, and *Nos2* expressed by microglia in NOD/ShiLtJ mice treated with vehicle or BMS202. n = 3 per group. **e,** counts of TH1 ($CD45^{+}CD11b^{-}CD4^{+}IFN\text{-}\gamma^{+}$), TH17 ($CD45^{+}CD11b^{-}CD4^{+}IL\text{-}17^{+}$), and regulatory IL-$10^{+}CD4^{+}$ T cells in the CNS of vehicle or BMS202 treated NOD/ShiLtJ mice. n = 6 per group. **f,** EAE development and timepoints indicated for the flow cytometric analysis of PD-L1 and PD-1 expression by glial cells. n = 20. **g,** representative scatterplots and quantification of PD-L1 expression by astrocytes, as well as PD-1 expression by microglia in the brain and spinal cord during EAE at the timepoints indicated in **(f)**. n = 3 per timepoint and group. **h,** immunostaining of PD-$L1^{+}$ astrocytes and PD-1^{+} microglia in brains of EAE mice. Data shown are representative of n = 12 fields from three distinct EAE brains. scale bar 50 µm. **i,** expression of microglial PD-1 expression in Cd11b-Scrmbl and Cd11b-Pdcd1 mice during EAE, quantified by flow cytometry. n = 3 per group. **j,** representative scatterplot depicting the gating strategy used to separate microglia from astrocytes in the astrocyte-microglia co-culture. **k,** relative expression (% of parent) of the activation marker CD44 and the B7 family members PD-L1 and PD-L2 by primary mouse astrocytes stimulated with IFN-γ. n = 4 per group. **l,** RT-qPCR analysis of *Bdnf*, *Cd86*, and *Il10* by primary mouse microglia following blockade of soluble PD-L1 by BMS202 or α-PD-L1. n = 4 per group. **m,** RT-qPCR analysis of *Bdnf* (n = 3 per group), *Cd86* (n = 4 per group), and *Il10* (n = 4 per group) by IFN-γ-activated primary mouse microglia following treatment with rmPD-L1. Unpaired t-test in **(e, f, k)**, One-way ANOVA with Tukey's multiple comparisons test in **(l, m)**. Data are shown as mean ± SEM in **(g)**. Data are shown as mean ± SD in if not indicated otherwise. Data are shown as mean with the 25th and 75th percentiles in **(d)**.
