# Supplementary material for: PD-L1 positive astrocytes attenuate inflammatory functions of PD-1 positive microglia in models of autoimmune neuroinflammation
Source: Nat Commun. 2023 Sep 9;14:5555. doi: 10.1038/s41467-023-40982-8 (PMC10492803; doi:10.1038/s41467-023-40982-8)
Supplement: Supplementary file 3 — Reporting Summary [file 41467_2023_40982_MOESM3_ESM.pdf]

nature portfolio

Corresponding author(s): Veit Rothhammer

Last updated by author(s): 04/07/2023

Reporting Summary

Nature Portfolio wishes to improve the reproducibility of the work that we publish. This form provides structure for consistency and transparency in reporting. For further information on Nature Portfolio policies, see our Editorial Policies and the Editorial Policy Checklist.

Statistics

For all statistical analyses, confirm that the following items are present in the figure legend, table legend, main text, or Methods section.

n/a	Confirmed	
☐	☒	The exact sample size (*n*) for each experimental group/condition, given as a discrete number and unit of measurement
☐	☒	A statement on whether measurements were taken from distinct samples or whether the same sample was measured repeatedly
☐	☒	The statistical test(s) used AND whether they are one- or two-sided<br>*Only common tests should be described solely by name; describe more complex techniques in the Methods section.*
☐	☒	A description of all covariates tested
☐	☒	A description of any assumptions or corrections, such as tests of normality and adjustment for multiple comparisons
☐	☒	A full description of the statistical parameters including central tendency (e.g. means) or other basic estimates (e.g. regression coefficient) AND variation (e.g. standard deviation) or associated estimates of uncertainty (e.g. confidence intervals)
☐	☒	For null hypothesis testing, the test statistic (e.g. *F*, *t*, *r*) with confidence intervals, effect sizes, degrees of freedom and *P* value noted<br>*Give P values as exact values whenever suitable.*
☒	☐	For Bayesian analysis, information on the choice of priors and Markov chain Monte Carlo settings
☒	☐	For hierarchical and complex designs, identification of the appropriate level for tests and full reporting of outcomes
☐	☒	Estimates of effect sizes (e.g. Cohen's *d*, Pearson's *r*), indicating how they were calculated

Our web collection on statistics for biologists contains articles on many of the points above.

Software and code

Policy information about availability of computer code

Data collection	NextSeq 500 (Illumina); Cytek Northern Lights; Cytek SpectroFlo (v3.0); BD FACSDIVA (v.8.0.1),<br>Zeiss Zen Black (v.2011).
Data analysis	Visualization and statistics were performed using GraphPad Prism v.9.5.1; Differential expression analysis of bulk RNA-Seq data was performed using R (v.4.2.2) and DESeq2 (v.1.38.0); Ingenuity Pathway Analysis, Ridgeplots were built using the Python v.3.5.0 package clusterProfiler v.4.4.4; ggplot2 (v.3.1.0); GSEA (v 6.3); MSigDB (v.6.2); Adobe Illustrator (1.0); Adobe Photoshop (v.24.0.1); FIJI (v.1.53); FlowJo (v.10.5.0); JASPAR (2022); OMIQ; Leica LAS (v4.13)

For manuscripts utilizing custom algorithms or software that are central to the research but not yet described in published literature, software must be made available to editors and reviewers. We strongly encourage code deposition in a community repository (e.g. GitHub). See the Nature Portfolio guidelines for submitting code & software for further information.

Data

Policy information about availability of data

All manuscripts must include a data availability statement. This statement should provide the following information, where applicable:

- Accession codes, unique identifiers, or web links for publicly available datasets
- A description of any restrictions on data availability
- For clinical datasets or third party data, please ensure that the statement adheres to our policy

The datasets generated during and/or analysed during the current study are available from the corresponding author on reasonable request. Sequencing data have been deposited into the Gene Expression Omnibus (GEO) under the SuperSeries accession number GSE239875. Spatial Transcriptomic data obtained from Hasel et al. is deposited under the Gene Expression Omnibus SuperSeries accession number GSE148612. Clinical data for patient samples can be found in Supplementary Table 1. All supplemental tables with processed data have been submitted with this manuscript. All other data and code that support the findings of this study are available from the corresponding author on reasonable request.

Human research participants

Policy information about studies involving human research participants and Sex and Gender in Research.

Reporting on sex and gender	Information on sex for the cohorts used in Figure 1 and 2 has been reported in Table 1. The sex-distrubution in these cohorts reflects the overall prevalence of Multiple Sclerosis. Patient material used for immunohistochemical analysis (Fig. 1d) was obtained from an untreated female MS patient.
Population characteristics	Information on sex, age, disease duration, disability (EDSS), and treatment can be found in Table 1.
Recruitment	Figure 1d: All control individuals and those with MS, or their next of kin, had given informed consent for autopsy and use of their brain tissue for research purposes from the MS center (Centre de Resources et de Compétences Sclérose en Plaques Pays de La Loire) of the Nantes University Hospital (ABM PFS13-003 “Collection sclérose en plaques”).<br><br>Figure 1h / Figure 2p: Patients were prespectively recruited in our neuroimmunology outpatient department for diagnostic procedures including CSF sampling. Patients were only included in this study if the diagnosis of multiple sclerosis was confirmed with detection of inflmmatory changes in the CSF including positive oligoclonal bands.<br>CSF was obtained from the Joint Biobank Munich in the framework of the German Biobank node.
Ethics oversight	This study was approved by the standing ethical committee (14/18S) at Technical University Munich. Written informed consent was obtained from every patient within the framework of the Biobank resources at the Department of Neurology at Technical University Munich, Germany, which is part of the national competence network Multiple Sclerosis.

Note that full information on the approval of the study protocol must also be provided in the manuscript.

Field-specific reporting

Please select the one below that is the best fit for your research. If you are not sure, read the appropriate sections before making your selection.

☒ Life sciences ☐ Behavioural & social sciences ☐ Ecological, evolutionary & environmental sciences

For a reference copy of the document with all sections, see nature.com/documents/nr-reporting-summary-flat.pdf

Life sciences study design

All studies must disclose on these points even when the disclosure is negative.

Sample size	N numbers range from n=3 to n=13, with n= individual mouse, based on previously published work with the same stimulation paradigm and readout (PMID: 36266581; PMID: 33888612)
Data exclusions	No data was excluded from the analysis
Replication	To ensure replication, all deep sequencing data were repeated in 3-6 mice per group per timepoint. For in vitro and in vivo experiments, at least three independent experiments were performed.
Randomization	Samples and mice were randomly allocated into biological groups.
Blinding	Experimenters were blinded to biological group during EAE scoring. Immunohistochemical analyses were performed blinded. For in vitro experiments, no blinding was required as it would not affect of the quantitive results (e.g. RT-qPCR analysis, Flow cytometric analysis, etc.).

Reporting for specific materials, systems and methods

We require information from authors about some types of materials, experimental systems and methods used in many studies. Here, indicate whether each material, system or method listed is relevant to your study. If you are not sure if a list item applies to your research, read the appropriate section before selecting a response.

Materials & experimental systems

n/a	Involved in the study
☐	☒ Antibodies
☐	☒ Eukaryotic cell lines
☒	☐ Palaeontology and archaeology
☐	☒ Animals and other organisms
☒	☐ Clinical data
☒	☐ Dual use research of concern

Methods

n/a	Involved in the study
☒	☐ ChIP-seq
☐	☒ Flow cytometry
☒	☐ MRI-based neuroimaging

Antibodies

Antibodies used

For Flow cytometry, the following antibodies and concentrations were used:
BV421-CD11b (1:200; Biolegend, #101235), BV480-CD11c (1:100; BD, #565627), BV510-F4/80 (1:100; Biolegend,# 123135), BV570-Ly6C (1:200; Biolegend,# 128029), BV605-CD80 (1:100; BD, #563052), BV650-CD56 (1:100; BD, #748098), BV650-CD8 (1:100; Biolegend, #100741), PE-eFlour610-CD140a (1:100; Thermo Fisher Scientific, #61140180), SuperBright780-MHCII (1:200; Thermo Fisher Scientific, #78532080), BV711-CD74 (1:200; BD, #740748), PE-CD45R/B220 (1:100; BD, #561878), PE-CD105 (1:100; Thermo Fisher Scientific, # 12-1051-82), PE-Ly6G (1:200; BioLegend, #127607), PE-CD140a (1:100; BioLegend, #135905), PE-O4 (1:100; Miltenyi, # 130117507), PE-Ter119 (1:100; Biolegend, #116207), PE-Ly6C (1:100; Biolegend, #128007), AF488-A2B5 (1:100; Novus Biologicals, #FAB1416G), PE-CD279 (1:100; Biolegend, #135206), PE-CD273 (1:100; Biolegend, #107205), AF488-CD274 (1:100; Thermo Fisher Scientific, #53598282), PE-Cy5-CD24 (1:200; Biolegend, #101811), PE-Cy7-CD31 (1:200; Thermo Fisher Scientific, #25031182), PE-Cy7-CD274 (1:100; Thermo Fisher Scientific, # 12598282), PerCP-eFlour710-CD86 (1:100; Thermo Fisher Scientific, #46086280), AF532-CD44 (1:100; Thermo Fisher Scientific, #58044182), PE-Cy5.5-CD45 (1:200; Thermo Fisher Scientific, #35045180), JF646-MBP (1:100; Novus Biologicals, #NBP2-22121JF646), APC-Cy7-Ly6G (1:200; Biolegend, #127623), AF700-O4 (1:200; R&D, #FAB1326N), BUV737-CD154 (1:100; BD, #741735), AF660-CD19 (1:100; Thermo Fisher Scientific, #606019380), APC/Fire810-CD4 (1:100; Biolegend, #100479), PE-eFlour610-iNOS (1:100; Thermo Fisher Scientific, #61592080), BV711-IL17a (1:100; Biolegend, #506941), PE-CCL2 (1:100; BD, #554443), FITC-CXCL12 (1:100; Thermo Fisher Scientific, # MA523547), PE-Cy5-FoxP3 (1:100; Thermo Fisher Scientific, #15-5773-82), PE-Cy7-IFNγ (1:100; Biolegend, #505826), PE PerCP-eFlour710-TNF (1:100; Thermo Fisher Scientific, #46732180), APC-GM-CSF (1:100; Thermo Fisher Scientific, #17733182), AF700-Ki67 (1:100; BioLegend, #652419), APC-eF780-Ki67 (1:100; Thermo Fisher Scientific, #47569882), APC-eF780-IFNγ (1:100; Fisher Scientific, #47731942). The following isotypes were used: AF488-Rat IgG2a Isotype Control (eBR2a) (1:100; Thermo Fisher Scientific, #53432180), PE-Cy7-Rat IgG2a Isotype Control (eBR2a) (1:100; Thermo Fisher Scientific, #25432182).

For immunohistochemistry, the following antibodies and concentrations were used:
PD-L1 (1:300; Biolegend, #329701), Alexa-Fluor-488-conjugated donkey anti–mouse IgG (1:500; Jackson Immunoresearch labs, #715545150), mouse anti-GFAP (1:500; Millipore; #MAB360), mouse anti GFAP-Cy3 (1:300; Sigma Aldrich, #C9205), rabbit anti-Iba1 (1:500; Abcam; #ab178846), rat anti-PD-L1 (1:200; Invitrogen; #14-5982-82), goat anti-PD-1 (1:300; R&D; #AF1021), donkey anti-rat IgG AF405 (1:500; Abcam; ab175670), donkey anti-rabbit IgG AF568 (1:500; Life Technologies; #A10042), donkey anti-goat IgG AF647 (1:500; Life Technologies; #A32849), donkey anti-mouse IgG AF488 (1:500; Life Technologies; #A21202).

For Chromatin immunoprecipitation, the following antibodies and concentrations were used:
rabbit anti-AHR (1:2000; Enzo Life Sciences, #BMLSA210), and rabbit IgG polyclonal isotype control (1:2000; Cell Signaling, #2975S).

Validation

All commercial antibodies in this study were validated, based on the manufacturers' websites. Antibodies were used for the appropriate animal host and application(s), as per the information provided on those websites:
1. BV421-CD11b (1:200; Biolegend, #101235):
https://www.biolegend.com/en-us/products/brilliant-violet-421-anti-mouse-human-cd11b-antibody-7163?GroupID=BLG10427
• Doni A, et al. 2015. J Exp Med. 212:905.
• Däbritz J, et al. 2016. Sci Rep. 6:20584.
• Chai Y, et al. 2016. PLoS One. 11: 0162853.
• Moderzynski K, et al. 2016. PLoS Negl Trop Dis. .
• Su Y, et al. 2022. J Hematol Oncol. 15:99.
• Hou X, et al. 2020. Cell Reports. 28(1):172-189.e7..
• Liu J, et al. 2019. Immunity. 50:600.
• Ilinykh PA, et al. 2020. Cell Host & Microbe. 27(6):976-991.
• Miller CM, et al. 2020. J Virol. 94:00:00.
• Li Q, et al. 2019. Neuron. 101:207.
• Klemm F, et al. 2020. Cell. 181(7):1643-1660.e17.
• Yan L, et al. 2021. Front Cell Neurosci. 15:750373.

2. BV480-CD11c (1:100; BD, #565627):
https://www.bdbiosciences.com/en-au/products/reagents/flow-cytometry-reagents/research-reagents/single-color-antibodies-ruo/bv480-mouse-anti-human-cd11c.566184
• Knapp W. W. Knapp .. et al., ed. Leucocyte typing IV : white cell differentiation antigens. Oxford New York: Oxford University Press; 1989:1-1182.
• Stacker SA, Springer TA. Leukocyte integrin P150,95 (CD11c/CD18) functions as an adhesion molecule binding to a counter-receptor on stimulated endothelium. J Immunol. 1991; 146(2):648-655. (Clone-specific: ELISA).
• Visser L, Shaw A, Slupsky J, Vos H, Poppema S. Monoclonal antibodies reactive with hairy cell leukemia. Blood. 1989; 74(1):320-325. (Immunogen: Immunocytochemistry (cytospins), Immunohistochemistry, Immunoprecipitation).

3. BV510-F4/80 (1:100; Biolegend, #123135):
https://www.biolegend.com/en-us/products/brilliant-violet-510-anti-mouse-f4-80-antibody-8934
• Schaller E, et al. 2002. Mol. Cell. Biol. 22:8035. (IHC)
• Stevceva L, et al. 2001. BMC Clin Pathol. 1:3. (IHC)
• Kobayashi M, et al.2008. J. Leukoc. Biol. 83:1354.
• Poeckel D, et al. 2009. J. Biol Chem. 284:21077.
• Glass AM, et al. 2013. J. Immunol. 190:4830.
• Koehm S, et al. 2007. J. Allergy Clin. Immunol. 120:570. (IHC)
• Rankin AL, et al. 2010. J. Immunol. 184:1526. (IHC)
• Sasi SP, et al. 2014. J Biol Chem. 289:14178.
• Thakus VS, et al. 2014. Toxicol Lett. 230:322.
• Watson NB, et al. 2015. J Immunol. 194:2796.
• Hirakawa H, et al. 2015. PLoS One. 10:119360.
• Radtke AJ, et al. 2020. Proc Natl Acad Sci U S A. 117:33455-65. (SB)

4. BV570-Ly6C (1:200; Biolegend, #128029):
https://www.biolegend.com/en-us/products/brilliant-violet-570-anti-mouse-ly-6c-antibody-7392
• Harsha Krovi S, et al. 2020. Nat Commun. 4.790277778.
• Sepe JJ, et al. 2022. JACC Basic Transl Sci. 7:915.
• Linnerbauer M, et al. 2022. Front Immunol. 12:800128.
• Li J, et al. 2020. Cancer Discov. .
• Wu X, et al. 2021. Elife. 10:.
• Li J, et al. 2020. Cancer Immunol Res. 0.529166667.
• Haase C, et al. 2022. Nat Methods. 19:1622.
• Stump CT, et al. 2021. Open Biol. 11:210245.
• Ajina R, et al. 2021. Cancer Immunol Res. 9:386.
• Hulsmans M et al. 2017. Cell. 169(3):510-522 .
• Li J, et al. 2018. Immunity. 49:178.
• , et al. 2021. Eur J Immunol. 51:2708.

5. BV605-CD80 (1:100; BD, #563052):
https://www.bdbiosciences.com/en-de/products/reagents/flow-cytometry-reagents/research-reagents/single-color-antibodies-ruo/bv605-hamster-anti-mouse-cd80.563052
• Bluestone JA. New perspectives of CD28-B7-mediated T cell costimulation. Immunity. 1995; 2(6):555-559. (Biology).
• Boussiotis VA, Gribben JG, Freeman GJ, Nadler LM. Blockade of the CD28 co-stimulatory pathway: a means to induce tolerance. Curr Opin Immunol. 1994; 6(5):797-807. (Biology).
• Hathcock KS, Laszlo G, Pucillo C, Linsley P, Hodes RJ. Comparative analysis of B7-1 and B7-2 costimulatory ligands: expression and function. J Exp Med. 1994; 180(2):631-640. (Biology).

6. BV650-CD56 (1:100; BD, #748098):
https://www.bdbiosciences.com/en-de/products/reagents/flow-cytometry-reagents/research-reagents/single-color-antibodies-ruo/bv650-rat-anti-mouse-cd56-ncam-1.748098
• Fujita T, Chen MJ, Li B, et al. Neuronal transgene expression in dominant-negative SNARE mice.. J Neurosci. 2014; 34 (50):16594-604. (Clone-specific: Fluorescence activated cell sorting).
• Li S, Nie EH, Yin Y, et al. GDF10 is a signal for axonal sprouting and functional recovery after stroke.. Nat Neurosci. 2015; 18 (12):1737-45. (Clone-specific: Fluorescence activated cell sorting).
• Rougon G, Deagostini-Bazin H, Hirn M, Goridis C. Tissue- and developmental stage-specific forms of a neural cell surface antigen linked to differences in glycosylation of a common polypeptide.. EMBO J. 1982; 1(10):1239-44. (Biology).

7. BV650-CD8 (1:100; Biolegend, #100741):
https://www.biolegend.com/en-us/products/brilliant-violet-650-anti-mouse-cd8a-antibody-7635
• Schädlich IS, et al. 2022. iScience. 25:104470.
• Flamar AL, et al. 2020. Immunity. 52(4):606-619.e6..
• Wiesner DL, et al. 2020. Cell Host Microbe. 614:27.
• Boyd DF, et al. 2020. Nature. 587:466.
• Kloepper J, et al. 2016. Proc Natl Acad Sci U S A. 113: 4476-4481.
• Schönberger K, et al. 2022. Cell Stem Cell. 29:131.
• Suresh R, et al. 2020. J Immunother Cancer. 8:.
• Arce Vargas F et al. 2018. Cancer cell. 33(4):649-663 .
• Piepke M, et al. 2021. J Neuroinflammation. 18:265.

• Sauter M, et al. 2022. iScience. 25:103677.
• Coleby R, et al. 2021. Clin Exp Rheumatol. :39.
• Abou-Hamad J, et al. 2022. iScience. 25:105524.

8. PE-eFlour610-CD140a (1:100; Thermo Fisher Scientific, #61140180):
https://www.thermofisher.com/antibody/product/CD140a-PDGFRA-Antibody-clone-APA5-Monoclonal/61-1401-80
9. SuperBright780-MHCII (1:200; Thermo Fisher Scientific, #78532080):
https://www.thermofisher.com/antibody/product/MHC-Class-II-I-Ab-Antibody-clone-AF6-120-1-Monoclonal/78-5320-80
10. BV711-CD74 (1:200; BD, #740748):
https://www.bdbiosciences.com/en-de/products/reagents/flow-cytometry-reagents/research-reagents/single-color-antibodies-ruo/bv711-rat-anti-mouse-cd74.740748
• Bertolino P, Rabourdin-Combe C. The MHC class II-associated invariant chain: a molecule with multiple roles in MHC class II biosynthesis and antigen presentation to CD4+ T cells. Crit Rev Immunol. 1996; 16(4):359-379. (Biology).
• Bikoff EK, Huang LY, Episkopou V, van Meerwijk J, Germain RN, Robertson EJ. Defective major histocompatibility complex class II assembly, transport, peptide acquisition, and CD4+ T cell selection in mice lacking invariant chain expression. J Exp Med. 1993; 177 (6):1699-1712. (Biology).
• Bodmer H, Viville S, Benoist C, Mathis D. Diversity of endogenous epitopes bound to MHC class II molecules limited by invariant chain. Science. 1994; 263(5151):1284-1286. (Biology).

11. PE-CD45R/B220 (1:100; BD, #561878):
https://www.bdbiosciences.com/en-de/products/reagents/flow-cytometry-reagents/research-reagents/single-color-antibodies-ruo/pe-rat-anti-mouse-cd45r-b220.561878
• Allman DM, Ferguson SE, Cancro MP. Peripheral B cell maturation. I. Immature peripheral B cells in adults are heat-stable antigenhi and exhibit unique signaling characteristics. J Immunol. 1992; 149(8):2533-2540. (Biology).
• Asensi V, Kimeno K, Kawamura I, Sakumoto M, Nomoto K. Treatment of autoimmune MRL/lpr mice with anti-B220 monoclonal antibody reduces the level of anti-DNA antibodies and lymphadenopathies. Immunology. 1989; 68(2):204-208. (Clone-specific).
• Ballas ZK, Rasmussen W. Lymphokine-activated killer cells. VII. IL-4 induces an NK1.1+CD8 alpha+beta- TCR-alpha beta B220+ lymphokine-activated killer subset. J Immunol. 1993; 150(1):17-30. (Biology).

12. PE-CD105 (1:100; Thermo Fisher Scientific, #12105182):
https://www.thermofisher.com/antibody/product/CD105-Endoglin-Antibody-clone-MJ7-18-Monoclonal/12-1051-82
13. PE-Ly6G (1:200; BioLegend, #127607):
https://www.biolegend.com/en-us/products/pe-anti-mouse-ly-6g-antibody-4777
• Lee T, et al. 2014. Mol Biol Cell. 25:583.
• Hernández-Santana YE, et al. 2020. Life Sci Alliance. 3:00.
• D'Alessandro G, et al. 2020. Eur J Immunol. 50:705.
• DeSouza-Vieira T, et al. 2020. Cell Rep. 33:108317.
• Bowling S, et al. 2020. Cell. 181(6):1410-1422.e27.
• Guo H, et al. 2020. Curr Protoc Immunol. 131:e107.
• Liang J, et al. 2021. Cancer Manag Res. 13:6977.
• Zhao D, et al. 2021. Innate Immun. 27:533.
• Zhou R, et al. 2022. EBioMedicine. 75:103762.
• Volmari A, et al. 2021. Hepatol Commun. 5:2104.
• Da Mesquita S, et al. 2021. Nature. 593:255.
• Combes F, et al. 2018. Neoplasia. 20:848.

14. PE-CD140a (1:100; BioLegend, #135905):
https://www.biolegend.com/en-us/products/pe-anti-mouse-cd140a-antibody-6253
• Gabitova-Cornell L, et al. 2020. Cancer Cell. 38(4):567-583.e11.
• Vercauteren Drubbel A, et al. 2021. Cell Stem Cell. .
• Zelic M, et al. 2021. Cell Reports. 35(6):109112.
• Buechler MB, et al. 2021. Nature. 593:575.
• Stoupa A, et al. 2018. EMBO Mol Med. 10:.
• Wagner G, et al. 2017. Sci Rep. 7:40881.
• Huang Z et al. 2017. Cell metabolism. 26(3):493-508 .
• Salzer MC et al. 2018. Cell. 175(6):1575-1590 .
• Chen M, et al. 2020. Sci Adv. 6:eaax9605.
• Wu R, et al. 2019. J Cell Mol Med. 24:1684.
• Biffi G, et al. 2018. Cancer Discov. 2:282.
• Cardot-Ruffino V, et al. 2020. Genesis. 58:e23359.

15. PE-O4 (1:100; Miltenyi, #130117507):
https://www.miltenyibiotec.com/DE-en/products/o4-antibody-anti-human-mouse-rat-o4.html
• G. Kantzer, C. et al. (2017) Anti-ACSA-2 defines a novel monoclonal antibody for prospective isolation of living neonatal and adult astrocytes. Glia (6) 65: 990 - 1004
• Bansal, R. et al. (1989) Multiple and novel specificities of monoclonal antibodies O1, O4, and R-mAb used in the analysis of oligodendrocyte development. J. Neurosci. Res. (4) 24: 548 - 557
• Zhang, S. C. (2001) Defining glial cells during CNS development. Nat. Rev. Neurosci. 2: 840 - 843
• Sommer, I. and Schachner, M. (1981) Monoclonal antibodies (O1 to O4) to oligodendrocyte cell surfaces: an immunocytological study in the central nervous system. Dev. Biol. 83: 311 - 327

• Jungblut, M. et al. (2012) Isolation and characterization of living primary astroglial cells using the new GLAST-specific monoclonal antibody ACSA-1. Glia (6) 60: 894 - 907
16. PE-Ter119 (1:100; Biolegend, #116207):
https://www.biolegend.com/en-us/products/pe-anti-mouse-ter-119-erythroid-cells-antibody-1867
• Guo H, Cooper S, Friedman A, et al. 2017. PLoS One. 10.1371/journal.pone.0150809.
• Grigsby SM, et al. 2021. Cancers (Basel). 13:.
• Furrer R, et al. 2021. Sci Adv. 7:eabi4852.
• Sun D, et al. 2021. Cell Stem Cell. .
• Schloss MJ, et al. 2022. Nat Immunol. 23:605.
• Xhima K, et al. 2020. Sci Adv. 6:eaax6646.
• Wong J, et al. 2015. Elife. 3: 07839.
• Hodzic D, et al. 2022. PLoS Biol. 20:e3001811.
• Hiraishi Y, et al. 2018. Sci Rep. 8:18052.
• Silva C, et al. 2019. Cell Physiol Biochem. 52:503.
• Papafragkos I, et al. 2022. Front Immunol. 13:889075.
• Endo Y, et al. 2020. FASEB J. 34:16086.

17. PE-Ly6C (1:100; Biolegend, #128007):
https://www.biolegend.com/en-us/products/pe-anti-mouse-ly-6c-antibody-4904
• Petersen B, et al. 2014. J Leukoc Biol. 95:809.
• Zuchtriegel G, et al. 2016. PLoS Biol. 14: 1002459.
• Jiang W, et al. 2017. Sci Rep. 7:6501.
• Gerwing M, et al. 2020. Mol Imaging Biol. 1.959027778.
• Tan L, et al. 2022. Biochem Biophys Rep. 32:101351.
• Zhou W, et al. 2019. Cell Syst. 0.597916667.
• Fu R, et al. 2020. Sci Rep. 10:1455.
• Radovanovic I, et al. 2014. J Immunol. 193:1290.
• Jiang W, et al. 2021. Oncol Lett. 22:625.
• Zhang YS, et al. 2018. Cancer Biol Ther. 19:735.
• Farsakoglu Y et al. 2019. Cell reports. 26(9):2307-2315 .
• Park JG, et al. 2021. iScience. 24(9):102941.

18. AF488-A2B5 (1:100; Novus Biologicals, #FAB1416G):
https://www.novusbio.com/products/a2b5-antibody-105_fab1416g
• Á Moreno-Gar, A Bernal-Chi, T Colomer, A Rodríguez-, C Matute, S Mato: Gene Expression Analysis of Astrocyte and Microglia Endocannabinoid Signaling during Autoimmune Demyelination Biomolecules, 2020;10(9):. 2020-01-01 [PMID: 32846891]
PE-CD279 (1:100; Biolegend, #135206):
https://www.biolegend.com/en-us/products/pe-anti-mouse-cd279-pd-1-antibody-6170
•
Harsha Krovi S, et al. 2020. Nat Commun. 4.790277778.
• Dong MB, et al. 2020. Cell. 178(5):1189-1204.e23..
• Spitsin S, et al. 2012. Clin Vaccine Immunol. 1.313888889.
• Renrick AN, et al. 2021. Front Immunol. 607044:12.
• Kaczanowska S, et al. 2021. Cell. 184(8):2033-2052.e21.
• Lin YN, et al. 2022. Oncoimmunology. 11:2027136.
• Zhang R, et al. 2021. Cell Mol Immunol. 18:1222.
• Hamilton JAG, et al. 2021. Aging Cell. 20:e13309.
• Sasse C, et al. 2022. Pathogens. 11:.
• Kim CJ, et al. 2018. Immunity. 49:1034.
• Delacher M, et al. 2021. Immunity. 54(4):702-720.e17.
• Stolp B, et al. 2022. Cell Rep. 38:110387.

19. PE-CD273 (1:100; Biolegend, #107205):
https://www.biolegend.com/en-us/products/pe-anti-mouse-cd273-b7-dc-pd-l2-antibody-2547
• Ochiai S, et al. 2014. J Immunol. 193:2504.
• Lakins MA, et al. 2018. Nat Commun. 9:948.
• Yonemitsu K, et al. 2022. Sci Rep. 12:12007.
• Rodriguez-García A, et al. 2021. Nat Commun. 12:877.
• Quatrini L, et al. 2018. Nat Immunol. 19:954.
• Pack AD, et al. 2021. Cell Reports. 36:109586.
• Fu Y, et al. 2020. Sci Rep. 10:9027.
• Ringel AE, et al. 2020. Cell. 183(7):1848-1866.e26.
• Garo LP, et al. 2019. Cell Rep. 28:3353.
• Abbott RK, et al. 2018. Immunity. 48:133.
• Jelinek D, et al. 2014. J Immunol. 192:3548.
• Pizzolla A, et al. 2016. PLoS One. 11: 0160407.

20. AF488-CD274 (1:100; Thermo Fisher Scientific, #53598282):
https://www.thermofisher.com/antibody/product/CD274-PD-L1-B7-H1-Antibody-clone-MIH5-Monoclonal/53-5982-82

21. PE-Cy5-CD24 (1:200; Biolegend, #101811):
https://www.biolegend.com/en-us/products/pe-cyanine5-anti-mouse-cd24-antibody-2936
• Springer T, et al. 1978. Eur. J. Immunol. 8:539. (WB)
• Crowley M, et al. 1989. Cell. Immunol. 118:108. (FA)
• Veillette A, et al. 1989. J. Exp. Med. 170:1671. (FA)
• Pandelakis A Flavell RA 1999 JEM 189:855 (FC, IHC)
• Liu JQ, et al. 2007 J. Immunol. 178:6227. (FC, IF)
• Chappaz S, et al. 2007. Blood doi:10.1182/blood-2007-02-074245. (FC)
• Rucci F, et al. 2010. Proc Natl Acad Sci USA. 107:3024. (FC)
• Teague TK, et al. 2010. Int Immunol. 22:387. (FC)
• Gracz AD, et al. 2010. Am J. Physiol Gastrointest Liver Physiol. 298:590. (FC)
• Chen CY, et al. 2008. Endocrinology. 10:1210. (FC, IHC)
• Qui Q, et al. 2010. J. Immunol. 184:1681. (FC)

22. PE-Cy7-CD31 (1:200; Thermo Fisher Scientific, #25031182):
https://www.thermofisher.com/antibody/product/CD31-PECAM-1-Antibody-clone-390-Monoclonal/25-0311-82
23. PE-Cy7-CD274 (1:100; Thermo Fisher Scientific, # 12598282):
https://www.thermofisher.com/antibody/product/CD274-PD-L1-B7-H1-Antibody-clone-MIH5-Monoclonal/12-5982-82
24. PerCP-eFlour710-CD86 (1:100; Thermo Fisher Scientific, #46086280):
https://www.thermofisher.com/antibody/product/CD86-B7-2-Antibody-clone-GL1-Monoclonal/46-0862-80
25. AF532-CD44 (1:100; Thermo Fisher Scientific, #58044182):
https://www.thermofisher.com/antibody/product/CD44-Antibody-clone-IM7-Monoclonal/58-0441-82
26. PE-Cy5.5-CD45 (1:200; Thermo Fisher Scientific, #35045180):
https://www.thermofisher.com/antibody/product/CD45-Antibody-clone-30-F11-Monoclonal/35-0451-80
27. JF646-MBP (1:100; Novus Biologicals, #NBP2-22121JF646):
https://www.novusbio.com/products/mbp-antibody-2h9_nbp2-22121jf646
28. APC-Cy7-Ly6G (1:200; Biolegend, #127623):
https://www.biolegend.com/en-us/products/apc-cyanine7-anti-mouse-ly-6g-antibody-6755
• Fleming TJ, et al. 1993. J. Immunol. 151:2399. (FC)
• Daley JM, et al. 2008. J. Leukocyte Biol. 83:1. (FC)
• Dietlin TA, et al. 2007. J. Leukocyte Biol. 81:1205. (FC)
• Daley J, et al. 2007. J. Leukocyte Biol. doi:10.1189. (Deplete)
• Tadagavadi RK, et al. 2010. J. Immunol. 185:4904.
• Sumagin R, et al. 2010. J. Immunol. 185:7057.
• Guiducci C, et al. 2010. J. Exp Med. 207:2931.
• Fujita M, et al. 2011. Cancer Res. 71:2664.
• Van Leeuwen, et al. 2008. Arterioscler. Thromb. Vasc. Biol. 28:84. (IHC)
• Kowanetz M, et al. 2010. P. Natl. Acad. Sci. USA 107:21248. [supplementary data] (IHC)
• Esbona K, et al. 2016. Breast Cancer Res. 18:35. (IHC)
• Wojtasiak M, et al. 2010. J. Gen. Virol. 91:2158. (FC, Deplete)

29. AF700-O4 (1:200; R&D, #FAB1326N):
https://www.rndsystems.com/products/oligodendrocyte-marker-o4-alexa-fluor-700-conjugated-antibody-o4_fab1326n
• Schachner, M. et al. (1981) Dev. Biol. 83:328.
• Bansal, R. et al. (1989) J. Neurosci. Res. 24:548.
• Bansal, R. and Pfeiffer, S.E. (1989) Proc. Natl. Acad. Sci. USA 86:6181.
• Gard, A. et al. (1995) Dev. Biol. 167:596.
• Reynolds, R. and Hardy, R. (1997) J. Neurosci. Res. 47:455.
• Ono, K. et al. (1997) J. Neurosci. Res. 48:212.
• Pang, Y. et al. (2000) J. Neurosci. Res. 62:510.
• Cai, Z. et al. (2001) Brain Res. 898:126.

30. BUV737-CD154 (1:100; BD, #741735):
https://www.bdbiosciences.com/en-de/products/reagents/flow-cytometry-reagents/research-reagents/single-color-antibodies-ruo/buv737-hamster-anti-mouse-cd154.741735
• Carbone E, Ruggiero G, Terrazzano G, et al. A new mechanism of NK cell cytotoxicity activation: the CD40-CD40 ligand interaction. J Exp Med. 1997; 185(12):2053-2060. (Biology).
• DeKruyff RH, Gieni RS, Umetsu DT. Antigen-driven but not lipopolysaccharide-driven IL-12 production in macrophages requires triggering of CD40. J Immunol. 1997; 158(1):359-366. (Biology).
• Dunn RJ, Luedecker CJ, Haugen HS, Clegg CH, Farr AG. Thymic overexpression of CD40 ligand disrupts normal thymic epithelial organization. J Histochem Cytochem. 1997; 45(1):129-141. (Biology).

31. AF660-CD19 (1:100; Thermo Fisher Scientific, #606019380):
https://www.thermofisher.com/antibody/product/CD19-Antibody-clone-eBio1D3-1D3-Monoclonal/606-0193-80
32. APC/Fire810-CD4 (1:100; Biolegend, #100479):
https://www.biolegend.com/en-us/products/apc-fire-810-anti-mouse-cd4-antibody-19552
• Dialynas DP, et al. 1983. J. Immunol. 131:2445. (Block, IP)
• Dialynas DP, et al. 1983. Immunol. Rev. 74:29. (IP, Deplete)
• Wu L, et al. 1991. J. Exp. Med. 174:1617. (Costim)

• Godfrey DI, et al. 1994. J. Immunol. 152:4783. (Block)
• Gavett SH, et al. 1994. Am. J. Respir. Cell. Mol. Biol. 10:587. (Deplete)
• Schuyler M, et al. 1994. Am. J. Respir. Crit. Care Med. 149:1286. (Deplete)
• Ghobrial RR, et al. 1989. Clin. Immunol. Immunopathol. 52:486. (Deplete)
• Israelski DM, et al. 1989. J. Immunol. 142:954. (Deplete)
• Zheng B, et al. 1996. J. Exp. Med. 184:1083. (IHC)
• Frei K, et al. 1997. J. Exp. Med. 185:2177. (IHC)
• Felix NJ, et al. 2007. Nat. Immunol. 8:388. (Block)
• Radtke AJ, et al. 2020. Proc Natl Acad Sci U S A. 117:33455-65. (SB)

33. PE-eFlour610-iNOS (1:100; Thermo Fisher Scientific, #61592080):
https://www.thermofisher.com/antibody/product/iNOS-Antibody-clone-CXNFT-Monoclonal/61-5920-80
34. BV711-IL17a (1:100; Biolegend, #506941):
https://www.biolegend.com/en-us/products/brilliant-violet-711-anti-mouse-il-17a-antibody-12030
• Kennedy J, et al. 1996. J. Interferon Cytokine Res. 16:611.
• Schubert D, et al. 2004. J. Immunol. 172:4503. (ICFC)
• Infante-Duarte C, et al. 2000. J. Immunol. 165:6107. (ICFC, ELISA Capture)
• Harrington LE, et al. 2005. Nature Immunol. doi:10.1038/ni1254. (ICFC, ELISA Capture)
• Nekrasova T, et al. 2005. J. Immunol. 175:2734. (ELISPOT Capture)
• Yen D, et al. 2006. J. Clin. Invest. 116:1310. (Neut)
• Ehirchiou D, et al. 2007. J. Exp. Med. 204:1519. (ICFC)
• Kang SG, et al. 2007. J. Immunol. 179:3724. (ICFC)
• Smith E, et al. 2008. J. Immunol. 181:1357. (Neut)
• Neufert C, et al. 2007. Eur. J. Immunol. 37:1809.
• Wang C, et al. 2009. Mucosal Immunol 2:173. (ICFC)
• Cui Y, et al. 2009. Invest. Ophth. Vis. Sci. 50:5811. (ICFC)

35. PE-CCL2 (1:100; BD, #554443):
https://www.bdbiosciences.com/en-de/products/reagents/flow-cytometry-reagents/research-reagents/single-color-antibodies-ruo/pe-hamster-anti-mouse-rat-mcp-1.554443
• Luo Y, Laning J, Hayashi M, Hancock PR, Rollins B, Dorf ME. Serologic analysis of the mouse beta chemokine JE/monocyte chemoattractant protein-1. J Immunol. 1994; 153(8):3708-3716. (Biology).
• Prussin C, Metcalfe DD. Detection of intracytoplasmic cytokine using flow cytometry and directly conjugated anti-cytokine antibodies. J Immunol Methods. 1995; 188(1):117-128. (Methodology).

36. FITC-CXCL12 (1:100; Thermo Fisher Scientific, # MA523547):
https://www.thermofisher.com/antibody/product/CXCL12-Antibody-clone-79018-Monoclonal/MA5-23547
37. PE-Cy5-FoxP3 (1:100; Thermo Fisher Scientific, #15577382):
https://www.thermofisher.com/antibody/product/FOXP3-Antibody-clone-FJK-16s-Monoclonal/15-5773-82
38. PE-Cy7-IFNγ (1:100; Biolegend, #505826):
https://www.biolegend.com/en-us/products/pe-cyanine7-anti-mouse-ifn-gamma-antibody-5865
• Abrams J, et al. 1992. Immunol. Rev. 127:5. (ELISA, Neut)
• Sander B, et al. 1993. J. Immunol. Meth. 166:201. (ELISA, Neut)
• Abrams J, et al. 1995. Curr. Prot. Immunol. John Wiley and Sons, New York. Unit 6.20. (ELISA, Neut)
• Yang X, et al. 1993. J. Immunoassay 14:129. (ELISA)
• Klinman D, et al. 1994. Curr. Prot. Immunol. John Wiley and Sons, New York. Unit 6.19. (ELISPOT)
• Sander B, et al. 1991. Immunol. Rev. 119:65. (IHC)
• Ferrick D, et al. 1995. Nature 373:255. (FC)
• Ko SY, et al. 2005. J. Immunol. 175:3309. (FC)
• Peterson KE, et al. 2000. J. Virol. 74:5363. (Neut)
• DeKrey GK, et al. 1998. Infect. Immun. 66:827. (Neut)
• Dzhagalov I, et al. 2007. J. Immunol. 178:2113. (ELISA)
• Lawson BR, et al. 2007. J. Immunol. 178:5366. (FC)

39. PE PerCP-eFlour710-TNF (1:100; Thermo Fisher Scientific, #46732180):
https://www.thermofisher.com/antibody/product/TNF-alpha-Antibody-clone-MP6-XT22-Monoclonal/46-7321-80
40. APC-GM-CSF (1:100; Thermo Fisher Scientific, #17733182):
https://www.thermofisher.com/antibody/product/GM-CSF-Antibody-clone-MP1-22E9-Monoclonal/17-7331-82
• Medina-Reyes EI, et al. 2015. Environ Res. 136:424.
• Guillaumond F, et al. 2015. PNAS. 112:2473.
• Sharma SK, et al. 2015. J Immunol. 194:5529.
• Rodero MP, et al. 2014. J. Invest. Dermatol. 7:1991-7.

41. AF700-Ki67 (1:100; BioLegend, #652419):
https://www.biolegend.com/en-us/products/alexa-fluor-700-anti-mouse-ki-67-antibody-10366
• Medina-Reyes EI, et al. 2015. Environ Res. 136:424.
• Guillaumond F, et al. 2015. PNAS. 112:2473.
• Sharma SK, et al. 2015. J Immunol. 194:5529.
• Rodero MP, et al. 2014. J. Invest. Dermatol. 7:1991-7.

42. APC-eF780-Ki67 (1:100; Thermo Fisher Scientific, # 47569882):
https://www.thermofisher.com/antibody/product/Ki-67-Antibody-clone-SolA15-Monoclonal/47-5698-82
43. APC-eF780-IFNγ (1:100; Fisher Scientific, #47731942):
https://www.thermofisher.com/antibody/product/IFN-gamma-Antibody-clone-4S-B3-Monoclonal/47-7319-42
44. AF488-Rat IgG2a Isotype Control (eBR2a) (1:100; Thermo Fisher Scientific, #53432180):
https://www.thermofisher.com/antibody/product/Rat-IgG2a-kappa-clone-eBR2a-Isotype-Control/53-4321-80
45. PE-Cy7-Rat IgG2a Isotype Control (eBR2a) (1:100; Thermo Fisher Scientific, #25432182):
https://www.thermofisher.com/antibody/product/Rat-IgG2a-kappa-clone-eBR2a-Isotype-Control/25-4321-82
46. PD-L1 (1:300; Biolegend, #329701):
https://www.biolegend.com/en-us/products/purified-anti-human-cd274-b7-h1-pd-l1-antibody-4373
• Brown J, et al. 2003. J. Immunol. 170:1257. (FC, IHC, Block)
• Radziewicz H, et al. 2007. J. Virol. 81:2545. (Block)
• Nakamoto N, et al. 2009. PLoS Pathog. 5:e1000313. (Block)
• Barsoum IB, et al. 2014. Cancer Res. 74:665.
• Haile, S et al. 2013. J. Immunol. 191:2829.
• RL M, et al. 2015. PNAS. 112:6506-6514.
• Mahoney KM, et al. 2015. Cancer Immunol. Res. 3:1308.

47. Alexa-Fluor-488-conjugated donkey anti–mouse IgG (1:500; Jackson Immunoresearch labs, #715545150):
https://www.jacksonimmuno.com/catalog/products/715-545-150
48. mouse anti-GFAP (1:500; Millipore; #MAB360):
https://www.merckmillipore.com/DE/de/product/Anti-Glial-Fibrillary-Acidic-Protein-Antibody-clone-GA5,MM_NF-MAB360
49. mouse anti GFAP-Cy3 (1:300; Sigma Aldrich, #C9205):
https://www.sigmaaldrich.com/DE/de/product/sigma/c9205
50. rabbit anti-Iba1 (1:500; Abcam; #ab178846):
https://www.abcam.com/products/primary-antibodies/iba1-antibody-epr16588-ab178846.html
• Saponara E et al. Loss of Hepatic Leucine-Rich Repeat-Containing G-Protein Coupled Receptors 4 and 5 Promotes Nonalcoholic Fatty Liver Disease. Am J Pathol 193:161-181 (2023).
• Bellut M et al. Delayed NLRP3 inflammasome inhibition ameliorates subacute stroke progression in mice. J Neuroinflammation 20:4 (2023).
• Liang Z et al. Long-Term High-Fat Diet Consumption Induces Cognitive Decline Accompanied by Tau Hyper-Phosphorylation and Microglial Activation in Aging. Nutrients 15:N/A (2023).
51. rat anti-PD-L1 (1:200; Invitrogen; #14-5982-82):
https://www.thermofisher.com/antibody/product/CD274-PD-L1-B7-H1-Antibody-clone-MIH5-Monoclonal/14-5982-82
52. goat anti-PD-1 (1:300; R&D; #AF1021):
https://www.rndsystems.com/products/mouse-pd-1-antibody_af1021
53. donkey anti-rat IgG AF405 (1:500; Abcam; ab175670):
https://www.abcam.com/products/secondary-antibodies/donkey-rat-igg-hl-alexa-fluor-405-preadsorbed-ab175670.html
• Schilpp C et al. TGF-ß1 increases permeability of ciliated airway epithelia via redistribution of claudin 3 from tight junction into cell nuclei. Pflugers Arch 473:287-311 (2021).
• Suzuki S et al. An mTORC1-dependent switch orchestrates the transition between mouse spermatogonial stem cells and clones of progenitor spermatogonia. Cell Rep 34:108752 (2021).
• Norden PR et al. Shear stimulation of FOXC1 and FOXC2 differentially regulates cytoskeletal activity during lymphatic valve maturation. Elife 9:N/A (2020).
54. donkey anti-rabbit IgG AF568 (1:500; Life Technologies; #A10042):
https://www.thermofisher.com/antibody/product/Donkey-anti-Rabbit-IgG-H-L-Highly-Cross-Adsorbed-Secondary-Antibody-Polyclonal/A10042

55. donkey anti-goat IgG AF647 (1:500; Life Technologies; #A32849):
https://www.thermofisher.com/antibody/product/Donkey-anti-Goat-IgG-H-L-Highly-Cross-Adsorbed-Secondary-Antibody-Polyclonal/A32849
56. donkey anti-mouse IgG AF488 (1:500; Life Technologies; #A21202):
https://www.thermofisher.com/antibody/product/Donkey-anti-Mouse-IgG-H-L-Highly-Cross-Adsorbed-Secondary-Antibody-Polyclonal/A-21202
57. rabbit anti-AHR (1:2000; Enzo Life Sciences, #BMLSA210):
https://www.enzolifesciences.com/BML-SA210/aryl-hydrocarbon-receptor-polyclonal-antibody/
• Ethanolic extract of Pyrrosia lingua (Thunb.) Farw. ameliorates OVX-induced bone loss and RANKL-induced osteoclastogenesis: S.A. Jang, et al.; Biomed. Pharmacother. 147, 112640 (2022), Abstract;
• STING is an intrinsic checkpoint inhibitor that restrains the TH17 cell pathogenic program: L.E.A. Damasceno, et al.; Cell Rep. 39, 110838 (2022), Abstract;
• The cyclin-dependent kinase inhibitor p27Kip1 interacts with the aryl hydrocarbon receptor and negatively regulates its transcriptional activity: D.J. Elson, et al.; FEBS Lett. 596, 2056 (2022), Abstract;

58. rabbit IgG polyclonal isotype control (1:2000; Cell Signaling, #2975S)
https://www.cellsignal.com/products/antibody-conjugates/rabbit-da1e-mab-igg-xp-isotype-control-alexa-fluor-488-conjugate/2975

Eukaryotic cell lines

Policy information about cell lines and Sex and Gender in Research

Cell line source(s)	HEK293T (Invitrogen, #K1711)<br>Human Astrocytes (ScienCell,#1800)
Authentication	Cell lines were authenticated prior to receipt by the commercial vendor using the STR-based method
Mycoplasma contamination	Cells tested negative for mycoplasma contamination by the commcercial vendor and upon receipt.
Commonly misidentified lines (See ICLAC register)	No commonly misidentified cell lines were used.

Animals and other research organisms

Policy information about studies involving animals; ARRIVE guidelines recommended for reporting animal research, and Sex and Gender in Research

Laboratory animals	C57BL/6J (The Jackson Laboratory, #000664). NOD/ShiLtJ (The Jackson Laboratory, #001976). Experiments were initiated in 8-12 week old mice.
Wild animals	The study did not involve wild animals.
Reporting on sex	EAE was induced in female mice only due to differences in susceptability and disease severity (PMID: 7517126; PMID: 15081249; PMID: 33190849). No sex-based analysis have been performed. For in vitro experiments, both sex were used.
Field-collected samples	Study did not involve field-collected samples
Ethics oversight	The animal studies were reviewed and approved by the standing ethical committees of the Bavarian State (Regierung von Oberbayern, AZ 55.2-2532.Vet_02-19-49; Regierung von Unterfranken, AZ 55.2.2-2532-2-1306).

Note that full information on the approval of the study protocol must also be provided in the manuscript.

Flow Cytometry

Plots

Confirm that:

- [x] The axis labels state the marker and fluorochrome used (e.g. CD4-FITC).
- [x] The axis scales are clearly visible. Include numbers along axes only for bottom left plot of group (a 'group' is an analysis of identical markers).
- [x] All plots are contour plots with outliers or pseudocolor plots.
- [x] A numerical value for number of cells or percentage (with statistics) is provided.

Methodology

Sample preparation

Isolation of cells from adult mouse CNS
Mice were perfused with cold 1× PBS and the CNS was isolated and mechanically diced using sterile razors. Brains and spinal cords were processed separately or pooled (if not indicated otherwise) and transferred into 5 ml of enzyme digestion solution consisting of 35.5 µl papain suspension (Worthington, #LS003126) diluted in enzyme stock solution (ESS) and equilibrated to 37°C. ESS consisted of 10 ml 10× EBSS (Sigma-Aldrich, #E7510), 2.4 ml 30% D(+)-glucose (Sigma-Aldrich, #G8769), 5.2 ml 1 M NaHCO3 (VWR, #AAJ62495-AP), 200 µl 500 mM EDTA (Thermo Fisher Scientific Scientific, #15575020), and 168.2 ml ddH2O, filter-sterilized through a 0.22-µm filter. Samples were shaken at 80 rpm for 30–40 min at 37°C. Enzymatic digestion was stopped with 1 ml of 10× hi ovomucoid inhibitor solution and 20 µl 0.4% DNase (Worthington, #LS002007) diluted in 10 ml inhibitor stock solution (ISS). 10× hi ovomucoid inhibitor stock solution contained 300 mg BSA (Sigma-Aldrich, #A8806) and 300 mg ovomucoid trypsin inhibitor (Worthington, #LS003086) diluted in 10 ml 1× PBS and filter sterilized using a 0.22-µm filter. ISS contained 50 ml 10× EBSS (Sigma-Aldrich, #E7510), 6 ml 30% D(+)-glucose (Sigma-Aldrich, #G8769), and 13 ml 1 M NaHCO3 (VWR, #AAJ62495-AP) diluted in 170.4 ml ddH2O and filter-sterilized through a 0.22-µm filter. Tissue was mechanically dissociated using a 5-ml serological pipette and filtered through a 70-µm cell strainer (Fisher Scientific, #22363548) into a fresh 50-ml conical tube. Tissue was centrifuged at 600g for 5 min and resuspended in 10 ml of 30% Percoll solution (9 ml Percoll (GE Healthcare Biosciences, #17-5445-01), 3 ml 10× PBS, 18 ml ddH2O). Percoll suspension was centrifuged at 600g for 25 min with no breaks. Supernatant was discarded and the cell pellet was washed once with 1× PBS, centrifuged at 500g for 5 min and prepared for downstream applications.

Spleens were mechanically dissected and dissociated by passing through a 100-µM cell strainer (Fisher Scientific, 10282631). Red blood cells were lysed with ACK lysing buffer (Life Technology, A10492-01) for 5 minutes and washed with 0.5% BSA and 2 mM EDTA at pH 8.0 in 1× PBS and prepared for downstream applications.

Instrument	Cytek Northern Lights (Cytek), CytoFLEX (Beckman Coulter), FACS Aria III (BD Biosciences)
Software	FlowJo (v10.5.1), SpectroFlo (v3.0)
Cell population abundance	Cell sorting was performed with the strictest purity setting (4-Way Purity); purity >95%. For cell population abundances see the respective figures. Population abundances are depicted as percent of live cells.
Gating strategy	Cells were gated according to the gating strategy provided in the Supplemental Figures. In brief, debris and duples were exluded using FSC/SSC. Next, live cells were identified by LIVE/DEAD-Aqua staining. CNS infiltrating cell types were identified based on their expression of the surface markers CD45 and CD11b. Positive / negative gates were defined based on the use of an unstained control, Fluorescence Minus One Control (FMO), or isotype control.

☒ Tick this box to confirm that a figure exemplifying the gating strategy is provided in the Supplementary Information.
